# Supplementary material for: Amis Pacilo and Yami Cipoho are not the same as the Pacific breadfruit starch crop—Target enrichment phylogenomics of a long-misidentified Artocarpus species sheds light on the northward Austronesian migration from the Philippines to Taiwan
Source: PLoS One. 2022 Sep 30;17(9):e0272680. doi: 10.1371/journal.pone.0272680 (PMC9524695; doi:10.1371/journal.pone.0272680)
Supplement: Attachment — Submitted filename: Response to Reviewers.pdf [file pone.0272680.s003.pdf]

Dear Dr. Reepmeyer,

We are submitting the revision of our manuscript (PONE-D-22-20826) titled "Amis *Pacilo* and Yami *Cipoho* are not the same as the Pacific breadfruit starch crop—Target enrichment phylogenomics of a long-misidentified *Artocarpus* species sheds light on the northward Austronesian migration from the Philippines to Taiwan" co-authored by co-authored by Chia-Rong Chuang, Chia-Lun Hsieh, Chi-Shan Chang, Chiu-Mei Wang, Danilo N. Tandang, Elliot M. Gardner, Lauren Audi, Nyree J. C. Zerega, and Kuo-Fang Chung.

For the journal requirements, below is our responses:

1. We have carefully checked and followed the PLoS ONE style templates.
2. We have removed all grant information from the Acknowledgement.
3. We have changed the Acknowledgement section and removed the grant information (Line 412–418).
4. We have deposited our data in GenBank (Bioproject no. PRJNA855987). The data is set to be release in 2026-07-05 and will be released immediately upon publication of the manuscript. For the purpose of review, please see the **Reviewer link** https://dataview.ncbi.nlm.nih.gov/object/PRJNA855987?reviewer=6mcs83vlkpsh7q7efe303ai465
5. We have stated in Line 202–205 that only oral permission is needed to sampled the studied species. If this does not fulfil the ethic requirement of PLoS ONE, please instruct and let me know how to proceed.
6. Figure 1 was drawn using QGIS and Illustrator by the authors and is not copyrighted.
7. Figure 3 has been removed to avoid violation of PLoS ONE copyright policy.
8. The references have been carefully reviewed to meet the PLoS ONE style. Reference 108 is added as this is an important reference to the statement neglected in the previous draft.

Below are our responses to Reviewer 1:

Reviewer #1: This paper uses new sequences from a relatively common tree taxa in Taiwan to revise its classification. It is sound research that will improve future studies through this clarification. Importantly, it also has implications for the history of Austronesians, a point that is well made in the manuscript.

Reply: We thank Reviewer #1 for the compliment.

Reviewer #1: I have only two minor suggestions. It might be useful to show a simplified phylogenetic tree of the taxa mentioned in the text for readers not familiar with these or their relation to one another. I would also revise the text of Figure 3 to make it clear there are unknown residents of the village in the photograph in addition to the Canadian visitor.

One typo: 1,300 AD should be 1300 AD.
Reply: We add a simplified phylogenetic tree of the taxa mentioned in the text in Fig. 3C. Because concern of the copyright issue, Figure 3 is removed in the revision. We also delete "," from all years mentioned in the text (Lines 75).

Reviewer #1: I searched for the underlying data on Genbank and the reported BioProject and could not find any under the identification listed. These should be confirmed as publicly available with publication of the study.
Reply: We have deposited our data in GenBank (Bioproject no. PRJNA855987). The data is set to be release in 2026-07-05 and will be released immediately upon publication of the manuscript. For the purpose of review, please see the **Reviewer link** https://dataview.ncbi.nlm.nih.gov/object/PRJNA855987?reviewer=6mcs83vlkpsh7q7efe303ai465

Below are our responses to Reviewer 2:
Reviewer #2: The authors perform a phylogenomic study of a Taiwanese plant taxa commonly referred to as 'breadfruit', building species trees from >500 gene trees and using the resulting phylogeny to examine multiple hypothesis concerning their taxanomic identity and their relationship with similar taxa on the neighbouring island of Lanyu. The data processing and phylogenomic methods appear to be robust and suitable for the present purpose (noting that I do not have direct experience with species tree reconstruction myself), and the authors clearly demonstrate that Taiwanese breadfruit fall within a single clade that is suggestive of a Lanyu origin, which is further attested by oral traditions of the local Indigenous peoples. Notably, the authors recognise the need for further research to help validate their claims, but overall I find the study to be well written and sufficiently informative to be deserving of publication in its current state.
Reply: We thank Reviewer #2 for the compliment.

Reviewer #2: Line 92-94: Fig. 1. caption - please state what the different coloured labels indicate.
Reply: We thank Reviewer #2 for the suggestions. Captions of Fig 1 (Line 93–97) and 3 (Line 293–297) have been modified to explain the meaning of colors used in both figures.

Reviewer #2: is this BWA aln or mem?
Reply: We thank Reviewer #2 for the questions. In the script of HybPiper pipeline [which are available on the HybPiper GitHub site (https://github.com/mossmatters/HybPiper)], it states that it is BWA mem used. As HybPiper is cited and freely available, we don't think it is necessary to specify this in the manuscript.

Reviewer #2: Line 261: 'to filtered out the column containing over 49% gaps' - should be 'to filter out the column ...'
Reply: We thank Reviewer #2 to spot this typo. We have corrected it (Line 262).

Reviewer #2: Line 291-294: Fig. 4. caption - please state what the bolded labels indicate.
Reply: We thank Reviewer #2 for the comments. Captions of Fig 1 (Line 93–97) and 3 (Line 293–297) have been modified to explain the meaning of colors and bold-faced used in both figures.
